# Correlation of Vaccine Responses

**DOI:** 10.3389/fimmu.2021.646677

**Published:** 2021-04-02

**Authors:** Petra Zimmermann, Nicole Ritz, Kirsten P. Perrett, Nicole L. Messina, Fiona R. M. van der Klis, Nigel Curtis

**Affiliations:** ^1^ Faculty of Science and Medicine, University of Fribourg, Fribourg, Switzerland; ^2^ Murdoch Children’s Research Institute, Parkville, VIC, Australia; ^3^ University of Basel, Basel, Switzerland; ^4^ The University of Melbourne, Parkville, VIC, Australia; ^5^ Royal Children’s Hospital Melbourne, Parkville, VIC, Australia; ^6^ National Institute for Public Health and the Environment, Bilthoven, Netherlands

**Keywords:** antibodies, immunization, titre, concentration, vaccination, live vaccines, pneumococcal

## Abstract

**Introduction:**

The humoral response to vaccinations varies widely between individuals. There is no data available on the correlation between responses to different vaccines. In this study, we investigated the correlation of antibody responses between routine vaccine antigens in infants.

**Methods:**

One and seven months after the 6-month vaccinations and one month after the 12-month vaccinations, antibody concentrations to diphtheria, tetanus, pertussis, polio (serotypes 1-3), *Haemophilus influenzae* type b (Hib), pneumococcus (13 serotypes), meningococcus C, measles, mumps and rubella were measured using fluorescent bead-based multiplex immune-assays. For the correlation of antibody responses, Spearman’s rank correlation coefficients (ρ) with 95% confidence intervals (CI) were calculated between responses to each vaccine antigen.

**Results:**

The correlation between concentrations of antibodies to the vaccinations ending at 6 months of age was higher one month compared to seven months after vaccination. The strongest correlations at both time points were observed between antibody responses to different polio serotypes, certain pneumococcal serotypes and between responses to diphtheria and pneumococcal (conjugated to a diphtheria toxoid) vaccine antigens. Correlation between responses to tetanus, Hib, pertussis, polio and other vaccine antigens were weak. The correlation between antibody responses to the 12-month vaccine antigens was weaker than to the 6-month vaccine antigens and there was a negative correlation between responses to measles, mumps, rubella vaccine and non-live vaccine antigens (meningococcus C, tetanus and Hib). There was only weak correlation between antibody responses to vaccines of the same type (e.g. conjugated polysaccharide or toxoid vaccines).

**Conclusion:**

Correlation between antibody responses to similar antigens in the same vaccine (such as different serotypes of a bacteria or virus), as well as responses to antigens conjugated to similar carrier proteins, are strong. In contrast, correlation between responses to other vaccines are weak. Measuring antibody responses to one or a few vaccine antigens therefore does not offer a reliable surrogate marker of responses to unrelated vaccines.

## Introduction

There are many different methods to quantify the immune response to vaccines, including measuring innate, humoral, cellular and cytokine responses. However, the most frequently used is the measurement of antibody concentrations. There is a wide variation between individuals in antibody responses induced by the same vaccine, and numerous intrinsic (including age, sex and genetics), external (including geographic location, family size and toxins) and behavioural (including nutrition, exercise, smoking and alcohol consumption) factors influence vaccine responses (1). The magnitude of response is also influenced by the type of vaccine. For example, live attenuated vaccines induce strong antibody responses, similar to infection with a wild-type pathogen, leading to induction of memory cells and often life-long protection. In contrast, inactivated, subunit or toxoid vaccines induce lower responses, requiring booster doses. The response to different subunit vaccines also varies: polysaccharide-protein conjugated vaccines have superior immunogenicity (including the induction of long-term protection) compared with polysaccharide vaccines which only induce short-lived T cell-independent antibody responses (2).

Currently, it is unknown whether there is a correlation between antibody responses to different vaccines. It is also unknown whether there is any consistency within individuals in the magnitude of antibody responses to all vaccines or to vaccines of a certain type (e.g. subunit, live or attenuated vaccines). In clinical practice, e.g. when investigating for primary immunodeficiency, measuring antibody responses to diphtheria, tetanus and pneumococcal vaccine antigens is common practice (3). When the vaccination status of an individual is unknown, the antibody response to tetanus is often used as a surrogate marker (4). However, there is no data available to show that the antibody response to one vaccine correlates with responses to other vaccines.

In this study, we investigated the correlation of antibody responses between routine vaccine antigens in infants.

## Methods

### Participants and Antibody Measurement

Participants were a subset of infants from a randomized controlled trial (The Melbourne Infant Study: BCG for Allergy and Infection Reduction (MIS BAIR)), which investigated whether Bacillus Calmette-Guérin vaccine (BCG) immunization given in the first 10 days of life protects against childhood infection, allergy and asthma. All infants were vaccinated according to the Australian National Immunisation Program: at birth: intramuscular hepatitis B (HepB) vaccine (H-B-Vax II Paediatric^®^ (*bioCSL*)); at 6 weeks, 4 months and 6 months of age: intramuscular combined diphtheria-tetanus-acellular pertussis (DTPa), HepB, polio (IPV), and *Haemophilus influenzae* type b (Hib) vaccine (Infanri*x^®^* hexa (*GlaxoSmithKline*)), intramuscular 13-valent conjugate pneumococcal vaccine (conjugated to CRM197, a diphtheria toxoid) (PCV13) (Prevenar1*3* ^®^ (*Wyeth*)), and oral rotavirus vaccine (RotaTeq^®^ (*Merck*)); at 12 months of age: subcutaneous measles-mumps-rubella (MMR) vaccine (Priorix^®^ (*GlaxoSmithKline*, Abbotsford, Victoria)) or M-M-R^®^ II (*Seqirus*, Parkville, Victoria)) and intramuscular combined meningococcal C (MenC) and Hib vaccine (conjugated to tetanus toxoid) (Menitorix^®^ (*GlaxoSmithKline*)). Vaccine records were obtained from individual immunization records and/or the Australian Immunisation Register.

From the subset of participants whose parent/guardian provided consent, blood samples were obtained in sodium-heparin tubes (S-monovette^®^ (*Sarstedt*)) during study visits at 7 and 13 months of age (one month after the administration of routine scheduled immunizations). Plasma was stored at -80°C until analysis. Only participants who had blood taken 28 ± 14 days after their 6-month and 12-month routine vaccines, respectively, were included in the final analysis. Additionally, persistence of antibodies was measured 7 months after the 6-month vaccines. For this, only participants who had their blood taken between 210 ± 14 days after their 6-month vaccinations were included, and, for the responses to tetanus and Hib only, participants who had not yet received their 12-month vaccinations, as Menitorix (Hib-MenC) given at 12 months of age includes a tetanus toxoid as carrier protein.

Blood samples were analyzed at the National Institute for Health and Environment, in Bilthoven, the Netherlands. Immunoglobulin (Ig) G antibodies against 26 vaccine antigens (diphtheria, tetanus, pertussis (pertussis toxin (PT), filamentous haemagglutinin (FHA), pertactin (PRN)), polio (types 1, 2, 3), Hib, pneumococcal ((Pn) serotypes 1, 3, 4, 5, 6A, 6B, 7F, 9V, 14, 18C, 19A, 19F, 23F), MenC, measles, mumps and rubella) were measured using fluorescent bead-based multiplex immune-assays (Luminex xMAP technology) (5–10). In all assays, an international or in-house reference, controls and blanks were included on each plate. All analyses were performed with a Bio-Plex 200 in combination with Bio-Plex manager software (Bio-Rad Laboratories, Hercules, CA).

### Statistical Analysis

The geometric mean concentration (GMC) for each vaccine antibody (immunoglobulin G) was calculated at one and seven months after vaccination. To investigate the correlation between antibody responses to different vaccines within the study cohort, pairwise Spearman’s rank correlation coefficients (ρ) with 95% confidence intervals (CI) were calculated between responses to each vaccine antigen. Correlations were categorized as follows: ‘very weak’ ρ = 0.00 - 0.19, ‘weak’ ρ = 0.20 - 0.39, ‘moderate’ ρ = 0.40 - 0.59, ‘strong’ ρ = 0.60 - 0.79 and ‘very strong’ ρ = 0.80 - 1.00.

Heatmaps were created to visualize the relationship between antibody responses to different vaccine antigens amongst all infants. Each individual’s response to a vaccine antigen was scaled by subtracting the mean of all responses to that vaccine antigen and dividing by the standard deviation [(individual vaccine response – mean vaccine response)/standard deviation]. A 5% significance level was used. All statistical analyses were done using R version 3.4.3.

### Ethics

Informed consent was obtained from participants’ parents or guardians. The study was approved by the Royal Children’s Hospital Human Research Ethics Committee (HREC, authorization (38124A).

## Results

In total, 91 participants had antibody responses to their 6-month vaccinations measured one month after vaccination and 141 participants seven months after vaccination (for Hib and tetanus, only the 34 participants who had not yet received the MenC-Hib vaccination were included at the second time point). Additionally, 148 participants had antibody responses to their 12-month vaccinations measured one month after vaccination. Thirty-three participants were included at both time points. The background characteristics of the participants are summarized in **Table 1**.

**Table 1:** Characteristics of study participants.

	Samples at 7 months of age for antibodies	Samples at 13 months of age for persistence of antibodies	Samples at 13 months of age for antibodies
	to primary course of vaccines ending at 6 months of age		to 12-month vaccines
	(n=91) n (%) or median (IQR)	(n=141) n (%) or median (IQR)	(n=148) n (%) or median (IQR)
Sex (male)	43 (47)	73 (52)	73 (49)
Gestational age (weeks)	39.5 (38.6-40.5)	39.2 (38.3-40.4)	39.2 (38.4-40.4)
Birth weight (kg)	3.48 (3.10-3.78)	3.37 (3.08-3.67)	3.41 (3.14-3.66)
Caesarean section	35 (38)	50 (35)	54 (36)
BCG-vaccinated MMR/MenC-Hib-vaccinated	45 (49) -	82 (58) 93 (66)	78 (53) 148 (100)
Maternal dTpa vaccination in pregnancy	46 (51)	81 (57)	69 (47)
Maternal influenza vaccination in pregnancy	55 (60)	79 (52)	88 (59)
Age at routine vaccination (days)			
- 6-week vaccines	45 (43-48)^1^	46 (43-50)	45 (43-48)
- 4-month vaccines	123 (117-127)	124 (103-130)	124 (120-131)
- 6-month vaccines	187 (181-193)	189 (184-195)	190 (185-202)
- 12-month vaccines	–	–	376 (369-383)
Interval between (days)			
- 6-month vaccines and 7-month blood sample	28 (21-37)	–	–
- 6-month vaccines and 13-month blood sample	–	211 (204-218)	–
- 12-month vaccines and 13-month blood sample	–	–	29 (22-36)
Age at blood sampling (days)	218 (208-227)	400 (394-411)	406 (397-414)

^1^age for one participant not available.

BCG, Bacille Calmette-Guérin; dTpa, diphtheria-tetanus-acellular pertussis vaccine; MMR, measles-mumps-rubella vaccine; MenC-Hib, meningococcal C-Haemophilus

influenzae type b.

**Figure 1:**
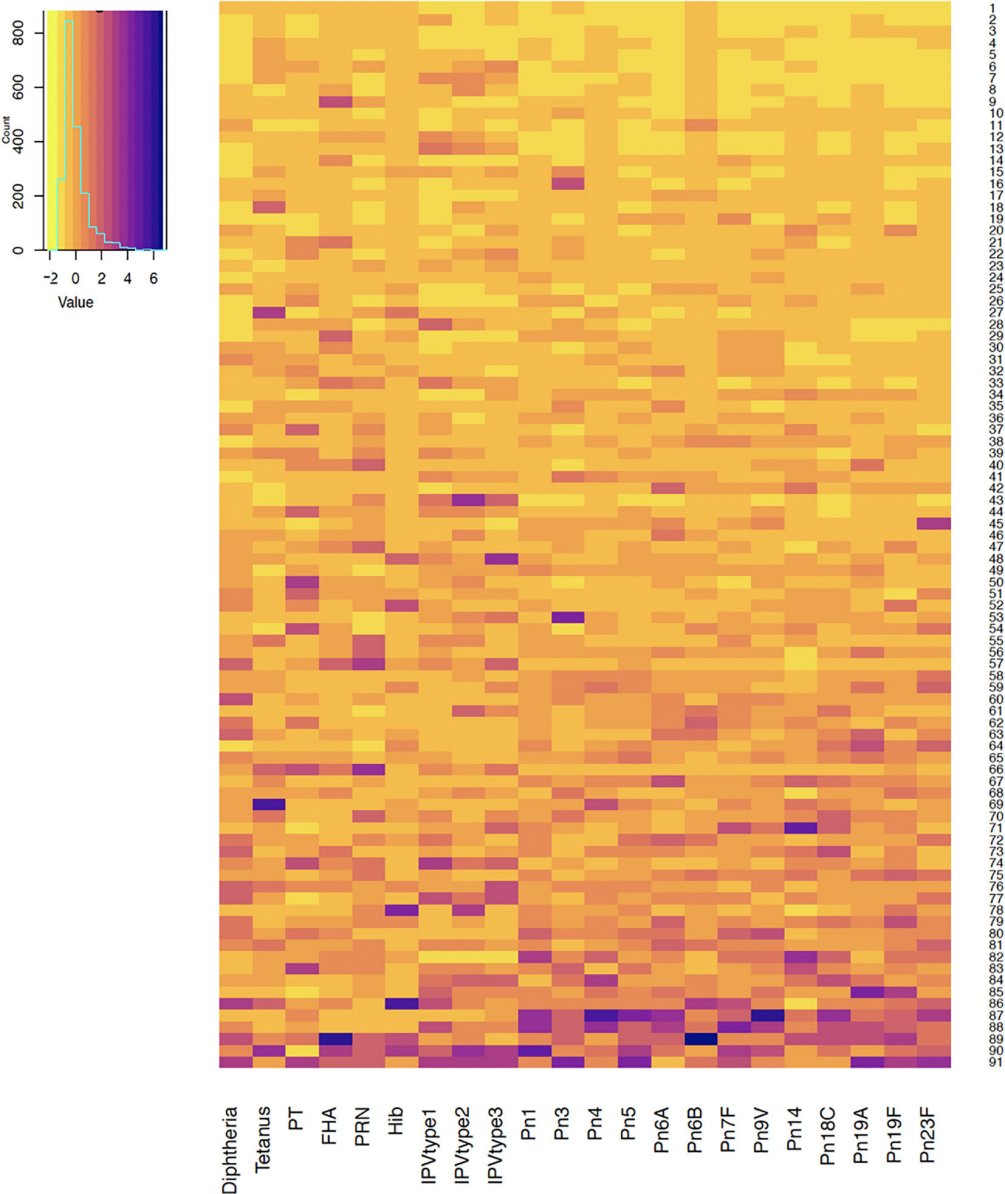
Antibody responses to vaccine antigens measured one month after the 6-month vaccinations (at 7 months of age) in 91 participants. Each row depicts one participant with the colour in each column representing the ‘scaled’ vaccine antibody response [(individual vaccine response - mean vaccine response)/standard deviation] in that individual. PT, pertussis toxin; FHA, filamentous haemagglutinin; PRN, pertactin; Hib, *Haemophilus influenzae* type b; IPV, polio; Pn, pneumococcal serotypes.

**Figure 2:**
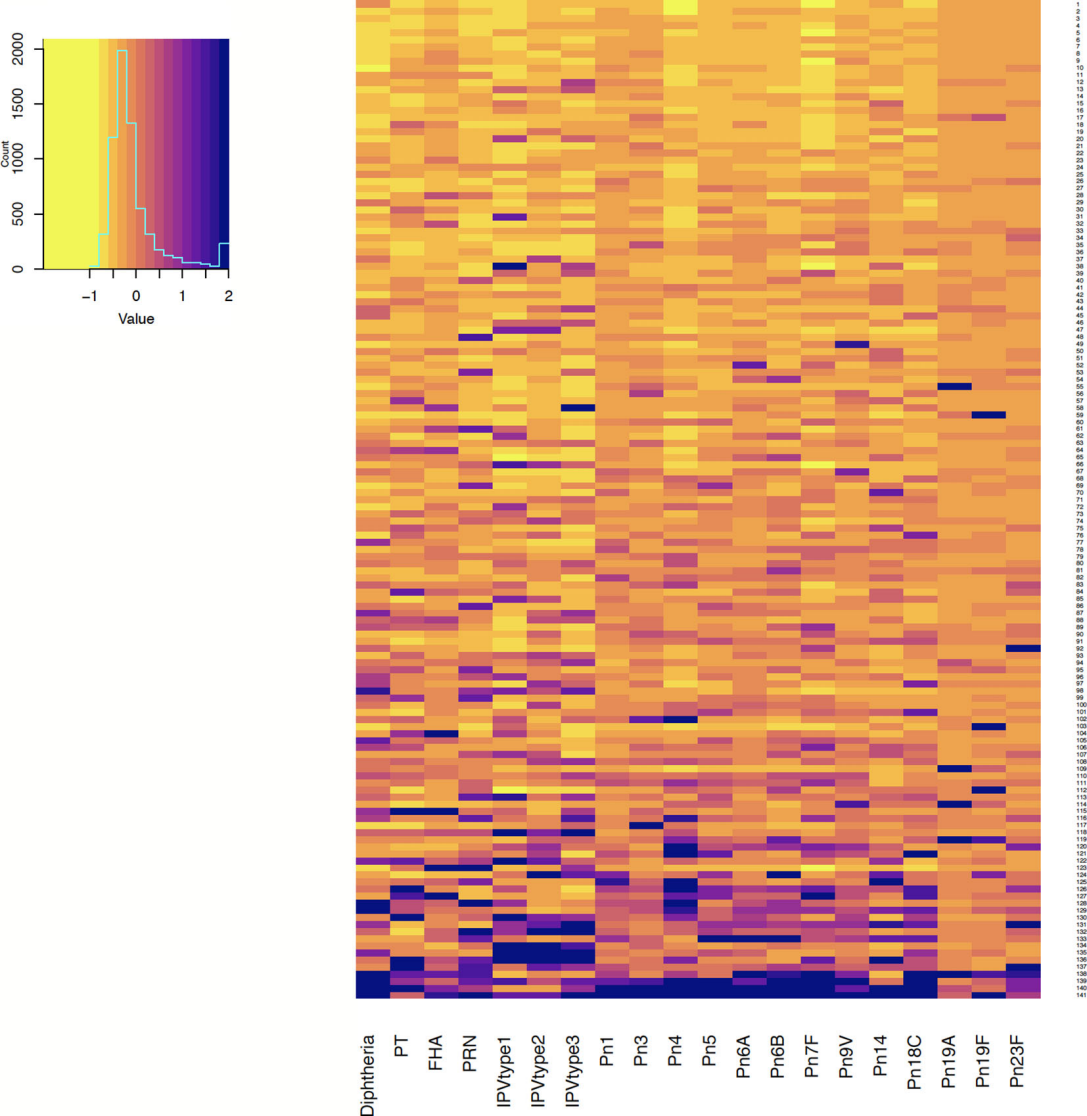
Antibody responses to vaccine antigens measured 7 months after 6-month vaccinations (at 13 months of age, for persistence of responses) in 141 participants. Each row depicts one participant with the colour in each column representing the ‘scaled’ [(individual vaccine response - mean vaccine response)/standard deviation] vaccine antibody response in that individual. PT, pertussis toxin; FHA, filamentous haemagglutinin; PRN, pertactin; IPV, polio; Pn, pneumococcal serotypes.

**Figure 3:**
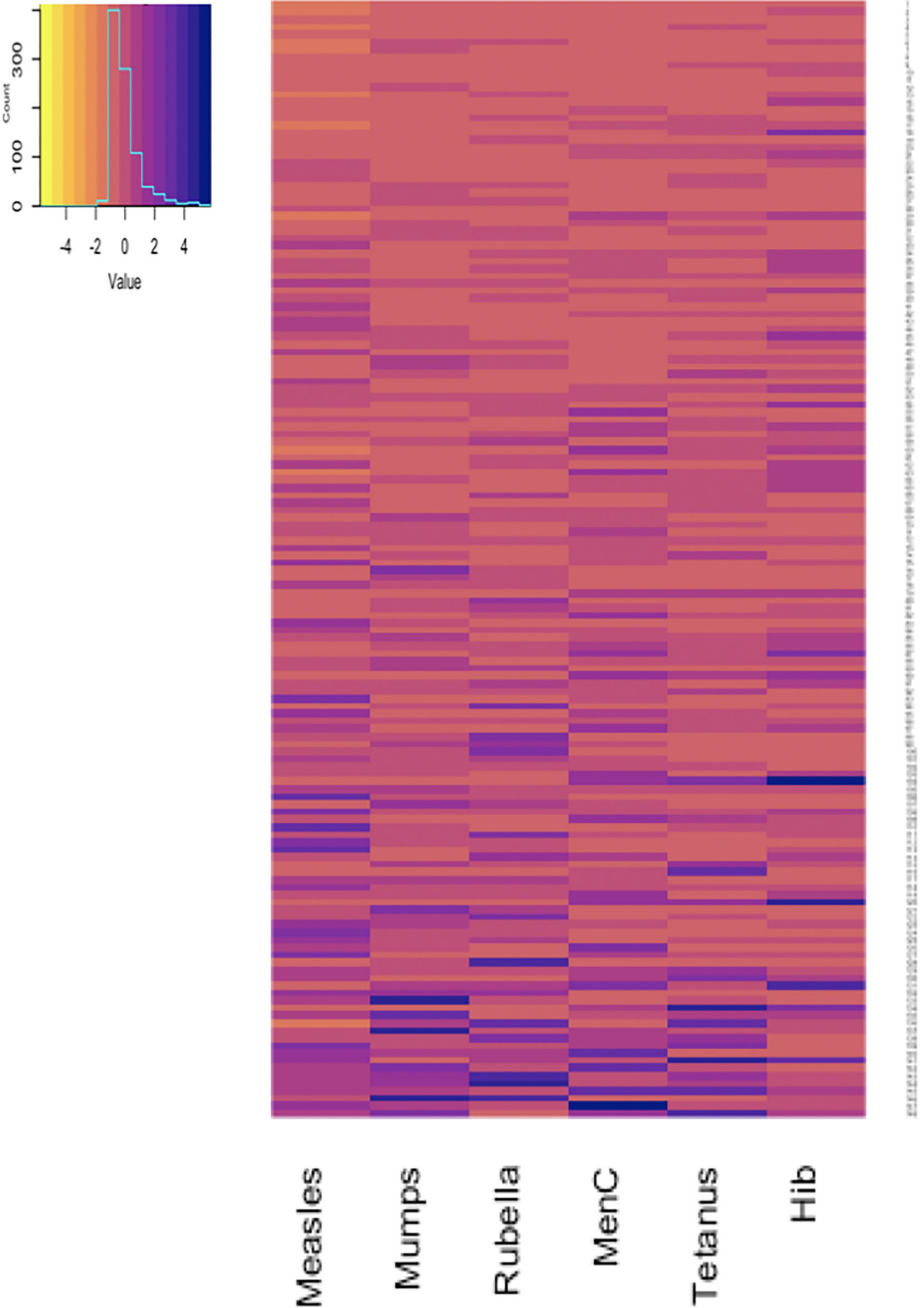
Antibody responses to 12-month vaccinations measured one month after vaccination in 148 participants. Each row depicts one participant with the colour in each column representing the ‘scaled’ [(individual vaccine response - mean vaccine response)/standard deviation] vaccine antibody response in that individual. MenC, Meningococcus C; Hib, *Haemophilus influenzae* type b.

Antibody responses in individuals one and seven months after 6-month vaccinations are shown in **Figures 1** and **2**. Antibody responses in individuals one month after the 12-month vaccinations are shown in **Figure 3**. Low responders are depicted at the top of the heatmap and high responders at the bottom.

### Correlation of Antibody Responses One Month After the 6-Month Vaccinations

**Figure 4:**
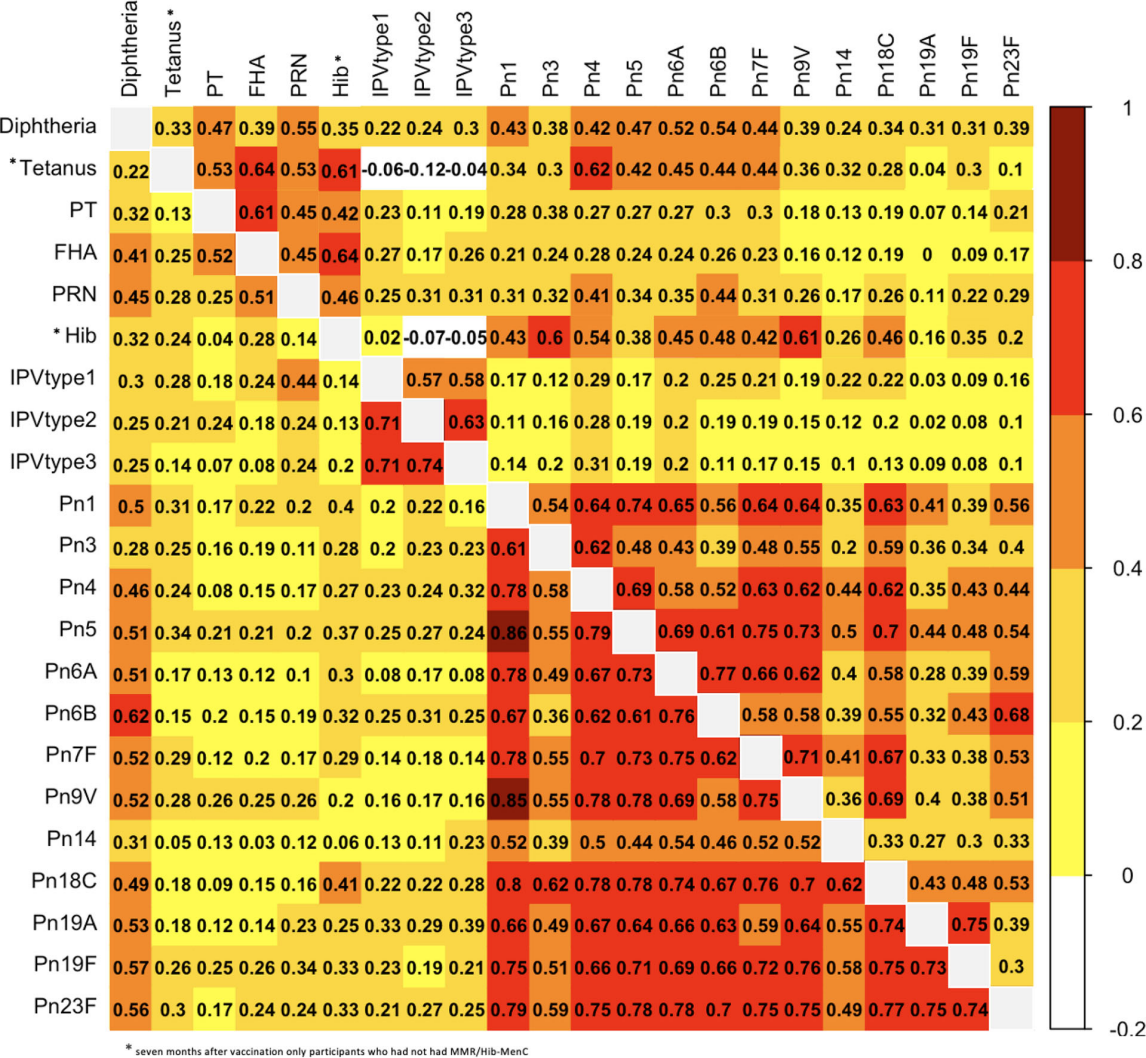
Correlation (expressed as Spearman correlation coefficients) for pairwise comparisons of antibody responses to the 6-month vaccinations measured at one (lower left) and seven (upper right) months after vaccination. PT, pertussis toxin; FHA, filamentous haemagglutinin; PRN, pertactin; Hib, *Haemophilus influenzae* type b; IPV, polio; Pn, pneumococcal serotypes.

Results of pairwise comparisons of antibody responses to the 22 vaccine antigens included in the 6-month vaccinations measured one month after vaccination are shown in **Figure 4** and **Supplementary Table 1**. One month after the 6-month vaccinations, ρ varied between 0.03 (between FHA and Pn14) and 0.86 (between Pn1 and Pn5). The strongest correlations were observed between antibody responses to different polio serotypes (strong) and different pneumococcal serotypes (moderate to very strong), with the exception of serotypes 3 and 14 (weak to strong). For the correlation between responses to different pneumococcal serotypes, the strongest correlations were observed for serotypes 1 (ρ = 0.52 - 0.86), 18C (ρ = 0.60 - 0.80) and 23F (ρ = 0.49 - 0.79). For antibody responses to pertussis, there was a moderate correlation between FHA and PRN (ρ = 0.51), and between FHA and PT (ρ = 0.52), but only a weak correlation between responses to PT and PRN (ρ = 0.25).

Antibody responses to diphtheria had moderate to strong correlations with pneumococcal serotypes, except pneumococcal serotypes 3 and 14 (weak). Additionally, antibody responses to diphtheria had moderate correlations with responses to FHA and PRN (ρ = 0.41 - 0.45), while the correlations between responses to other vaccine antigens were weak. Antibody responses to tetanus, Hib and polio serotypes 1-3 had very weak to weak correlations with responses to all the other vaccine antigens.

### Correlation of Antibody Responses Seven Months After the 6-Month Vaccinations

Results of pairwise comparisons between antibody responses to the 22 vaccine antigens in the 6-month vaccinations measured seven months after vaccination are shown in **Figure 4** and **Supplementary Table 1**. Seven months after the 6-month vaccinations, correlations between antibody responses were weaker than one month after the vaccinations, except for correlations between FHA and PT, tetanus and FHA, tetanus and Hib, and Hib and FHA for which correlations were stronger at seven months compared with one month after vaccination (0.61 vs 0.52, 0.64 vs 0.41, 0.61 vs 9.24, 0.64 vs 0.28) (**Figure 1** and **Supplementary Table 1**). The stronger correlations between tetanus and Hib and those to certain other antigens might be explained by the lower number of participants for these antigens (only participants who had not had MMR/Hib-MenC were included). Overall, 7 months after vaccination, ρ varied between -0.19 (between tetanus and polio serotype 2) and 0.77 (between Pn6A and Pn6B). The strongest correlations were observed between antibody responses to different pneumococcal serotypes (mostly moderate to strong with the exception of responses to serotypes 14, 19A and 19F and other serotypes, for which correlations were weak to moderate (except for the strong correlation between responses to 19A and 19F)). Moderate to strong correlations were also observed between responses to different polio serotypes and between different pertussis antigens.

Correlations between antibody responses to pneumococcal serotypes and diphtheria were weak to moderate. Antibody responses to tetanus correlated strongly with responses to FHA and Hib, but only weakly to moderately with responses to all the other antigens. The correlation between responses to tetanus and polio serotypes 1-3 were weakly negative, as was the correlation between responses to polio serotypes 2-3 and Hib. All other correlations between responses to polio and other vaccine antigens were very weak to weak.

### Correlation of Antibody Responses One Month After the 12-Month Vaccinations

**Figure 5:**
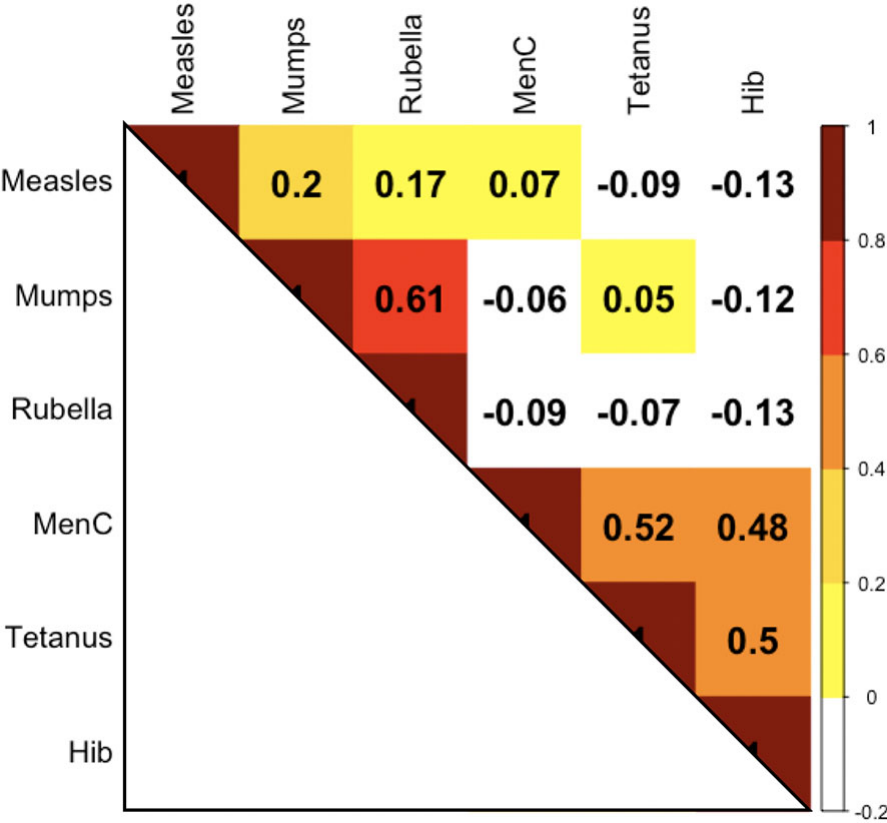
Correlation (expressed as Spearman correlation coefficients) for pairwise comparisons of antibody responses to the 12-month vaccinations at 13 months of age MenC, Meningococcus C; Hib, *Haemophilus influenzae* type b.

Results of pairwise comparisons of antibody responses to the 6 vaccine antigens measured one month after the 12-month vaccination are shown in **Figure 5** and **Supplementary Table 2**.

Overall, correlations between antibody responses to the 12-month vaccinations were weaker than those between responses to the 6-month vaccinations (**Figure 2** and **Supplementary Data Table 2**). There was a high correlation between antibody responses to mumps and rubella (ρ = 0.61) and moderate correlations between responses to tetanus and MenC (ρ = 0.52), between tetanus and Hib (ρ = 0.5), and between MenC and Hib (ρ = 0.48). Almost all of the correlations between the live-vaccines measles, mumps, rubella and non-live vaccines (MenC, tetanus, Hib) were weakly negative.

## Discussion

In this study, we observed a wide variation in antibody responses to vaccines and that overall, correlations between responses to different vaccines are weak. Strong correlations were found only between similar antigens (e.g. pneumococcal or polio serotypes) or between antigens that were conjugated to a protein carrier of the same or similar antigen. For example, in the conjugate pneumococcal vaccine, pneumococcal antigens are conjugated to CRM197, which is a diphtheria toxoid. This likely explains the moderate correlations between antibody responses to pneumococcal serotypes and diphtheria toxoid after the 6-month vaccinations. Similarly, for the combined Hib and MenC vaccine, both antigens are conjugated to a tetanus toxoid, which likely explains the moderate correlation between antibody responses to tetanus and Hib, and between tetanus and MenC observed after the 12-month vaccinations. However, after the 6-month vaccinations, there was only a weak correlation between the response to tetanus and Hib despite the latter being conjugated to tetanus toxoid. In contrast, this correlation was strong seven months after vaccination. We cannot explain this difference between immediate immune response and persistence of antibodies.

Despite being administered in the same vaccine, there were only weak correlations between antibody responses to tetanus, pertussis, polio and Hib antigens. Interestingly, we found negative correlations between responses to the live-vaccines measles, mumps and rubella, and non-live vaccines (MenC, tetanus, Hib). The finding is not easily explained as several studies have shown that simultaneous or delayed administration of MMR does not influence antibody concentrations to non-live vaccines (11–14).

Although infants were vaccinated simultaneously with two vaccines at each time point (which excludes confounding factors such as such as age, nutritional status, microbiota, time of day of administration etc.), correlations might be explained by the influence of factors such as adjuvants in the vaccines, vaccination site, needle size etc. Nevertheless, the vaccines in the study were given exactly as in clinical practice. Therefore, our findings are relevant to patient care. As the correlation between responses to different pneumococcal serotypes is strong, it may not be necessary to measure multiple responses to all serotypes. Serotypes which could potentially be used as a surrogate marker for response to pneumococcal vaccination are 18C, 1 and 23F. Responses to serotypes 14 and 3 should not be used as a surrogate, as responses to these antigens have the weakest correlations with responses to other serotypes. These findings are consistent with a recent study that reported correlations between 0.43 and 0.67 for responses to PCV13 (15). This study also observed the lowest correlations for antibody responses against pneumococcal serotype 14 and other serotypes (15).

Antibody responses to pneumococcal antigens may also be used to predict responses to diphtheria vaccination, especially when measured one month after vaccination, but not for responses to other vaccine antigens as other correlations were weak. Similarly, our finding of a strong correlation between antibody responses to different polio serotypes one month after vaccination means measuring responses to one serotype can be used to predict responses to other polio serotypes.

In contrast, antibody responses to pertussis, tetanus, polio and Hib were only weakly correlated with other vaccines responses, and therefore should not be used as surrogates for vaccine immunity. Notably, our results indicate that responses to non-live vaccines do not predict responses to live-vaccines.

As routine vaccinations include the administration of many different vaccine types, including toxoid, conjugated polysaccharide, inactivated and live vaccines, it is difficult to evaluate any consistency in the magnitude of antibody responses to vaccines of a certain type. Correlation between antibody responses to toxoid vaccines (diphtheria and tetanus) were weak at both one and seven months after vaccinations. Correlation between antibody responses to polysaccharide vaccines (Hib and pneumococcus) were mostly very weak to weak (with a few exceptions). Correlation between antibody responses to inactivated vaccine (polio) and purified proteins (pertussis) could not be compared with other vaccines of the same type. Furthermore, except the strong correlation that was found between responses to mumps and rubella, correlations between live vaccines were weak.

The findings of this study are relevant to clinical practice. As measuring antibody responses to one antigen cannot be used to predict responses to other antigens, this needs to be considered when using vaccine responses to investigate for primary immunodeficiency.

The strengths of this study include the broad range of measured antibody responses and the novel approach to comparing responses between different vaccine antigens. The limitations include the fact that only antibody concentration and not function was measured, that not all responses were measured exactly four weeks after vaccination, that responses to HepB vaccination were not measured (as this antigen is not included in the multiplex assay and there was insufficient sample volume for standard assays), and that the number of participants who were included at both time points was too small to allow longitudinal analysis.

In summary, the correlation between responses to similar antigens in the same vaccine (such as different serotypes of a bacteria or virus), as well as responses to antigens conjugated to similar carrier proteins is strong. In contrast, the correlation between responses to other vaccines is weak. Measuring antibody responses to one or a few vaccine antigens therefore does not offer a reliable surrogate marker of responses to unrelated vaccines.

## Data Availability Statement

The raw data supporting the conclusions of this article will be made available by the authors, without undue reservation.

## Ethics Statement

The studies involving human participants were reviewed and approved by Royal Children’s Hospital Human Research Ethics Committee [HREC, authorization (38124A)]. Written informed consent to participate in this study was provided by the participants’ legal guardian/next of kin.

## Author Contributions 

PZ, NR, KP, and NC were responsible for designing the study. NM was responsible for selecting the samples. FK was responsible for analyzing the samples. PZ performed the statistical analysis, which was supervised by NR, KP, and NC. PZ drafted the initial manuscript. All authors contributed to the article and approved the submitted version.

## Funding

This work was supported by the Australian National Health and Medical Research Council (NHMRC) (grants GNT1051228 and GNT1099680). KP is supported by a Melbourne Children’s Clinician-Scientist Fellowship.

## Conflict of Interest

The authors declare that the research was conducted in the absence of any commercial or financial relationships that could be construed as a potential conflict of interest.

## Supplementary Material

Supplementary Table 1 Correlation (Spearman correlation coefficients with 95% confidence intervals and p-values) for pairwise comparisons of antibody responses to the 6-month vaccinations measured one (lower left) and seven (upper right) months after vaccination. *seven months after vaccination only participants who had not had MMR/Hib-MenC.

Supplementary Table 2 Correlation (expressed as Spearman correlation coefficients with 95% confidence interval and p-values) for pairwise comparisons of antibody responses to the12-month vaccinations measured one month after vaccination.
