# Supplementary material for: Correlation of Vaccine Responses
Source: Front Immunol. 2021 Apr 2;12:646677. doi: 10.3389/fimmu.2021.646677 (PMC8050335; doi:10.3389/fimmu.2021.646677)
Supplement: Supplementary Table 1 — Correlation (Spearman correlation coefficients with 95% confidence intervals and p-values) for pairwise comparisons of antibody responses to the 6-month vaccinations measured one (lower left) and seven (upper right) months after vaccination. *seven months after vaccination only participants who had not had MMR/Hib-MenC. [file DataSheet_1.docx]

**Supplementary Table 1** Correlation (Spearman correlation coefficients with 95% confidence intervals and p-values) for pairwise comparisons of antibody responses to the 6-month vaccinations measured one (lower left) and seven (upper right) months after vaccination

|  | Diphtheria | Tetanus* | PT | FHA | PRN | Hib* | IPV type 1 | IPV type 2 | IPV type 3 | Pn 1 | Pn 3 | Pn4 | Pn 5 | Pn 6A | Pn 6B | Pn 7F | Pn 9V | Pn 14 | Pn 18C | Pn 19A | Pn 19F | Pn 23F |
| --- | --- | --- | --- | --- | --- | --- | --- | --- | --- | --- | --- | --- | --- | --- | --- | --- | --- | --- | --- | --- | --- | --- |
|  |  |  |  |  |  |  |  |  |  |  |  |  |  |  |  |  |  |  |  |  |  |  |
| Diphtheria |  | 0.33  (-0.01-0.60)  0.06 | **0.47**  **(0.32-0.59)**  **<0.01** | **0.39**  **(0.24-0.52)**  **<0.01** | **0.55**  **(0.43-0.66)**  **<0.01** | **0.35**  **(0.01-0.61)**  **0.04** | **0.22**  **(0.06-0.38)**  **<0.01** | **0.24**  **(0.08-0.39)**  **<0.01** | **0.30**  **(0.15-0.45)**  **<0.01** | **0.43**  **(0.28-0.55)**  **<0.01** | **0.38**  **(0.22-0.51)**  **<0.01** | **0.42**  **(0.28-0.55)**  **<0.01** | **0.47**  **(0.22-0.59)**  **<0.01** | **0.52**  **(0.38-0.63)**  **<0.01** | **0.54**  **(0.41-0.65)**  **<0.01** | **0.44**  **(0.29-0.56)**  **<0.01** | **0.39**  **(0.24-0.52)**  **<0.01** | **0.24**  **(0.08-0.39)**  **<0.01** | **0.34**  **(0.19-0.48)**  **<0.01** | **0.31**  **(0.15-0.45)**  **<0.01** | **0.31**  **(0.15-0.45)**  **<0.01** | **0.39**  **(0.24-0.52)**  **<0.01** |
| Tetanus* | **0.22**  **(0.02-0.41)**  **0.03** |  | **0.54**  **(0.24-0.74)**  **<0.01** | **0.64**  **(0.38-0.80)**  **<0.01** | **0.53**  **(0.23-0.74)**  **<0.01** | **0.61**  **(0.35-0.79)**  **<0.01** | -0.06  (-0.39-0.28)  0.72 | -0.12  (-0.44-0.23)  0.51 | -0.04  (-0.38-0.30)  0.81 | **0.34**  **(0.00-0.61)**  **0.05** | 0.30  (-0.04-0.58)  0.08 | **0.62**  **(0.35-0.79)**  **<0.01** | **0.42**  **(0.09-0.66)**  **0.01** | **0.45**  **(0.13-0.68)**  **<0.01** | **0.44**  **(0.12-0.68)**  **<0.01** | **0.44**  **(0.12-0.68)**  **<0.01** | **0.36**  **(0.03-0.62)**  **0.04** | 0.32  (-0.02-0.59)  0.07 | 0.28  (-0.07-0.56)  0.11 | 0.04  (-0.30-0.37)  0.83 | 0.30  (-0.05-0.58)  0.09 | 0.10  (-0.24-0.43)  0.55 |
| PT | **0.32**  **(0.12-0.49)**  **<0.01** | 0.13  (-0.08-0.33)  0.22 |  | **0.61**  **(0.49-0.70)**  **<0.01** | **0.45**  **(0.31-0.57)**  **<0.01** | **0.42**  **(0.10-0.67)**  **0.01** | **0.23**  **(0.07-0.38)**  **<0.01** | 0.11  (-0.06-0.27)  0.21 | **0.19**  **(0.03-0.35)**  **0.02** | **0.28**  **(0.12-0.43)**  **<0.01** | **0.38**  **(0.22-0.51)**  **<0.01** | **0.27**  **(0.11-0.41)**  **<0.01** | **0.27**  **(0.11-0.41)**  **<0.01** | **0.27**  **(0.11-0.42)**  **<0.01** | **0.30**  **(0.14-0.44)**  **<0.01** | **0.30**  **(0.14-0.44)**  **<0.01** | **0.18**  **(0.01-0.33)**  **0.03** | 0.13  (-0.04-0.29)  0.13 | **0.19**  **(0.03-0.35)**  **0.02** | 0.07  (-0.09-0.24)  0.38 | 0.14  (-0.02-0.30)  0.09 | **0.21**  **(0.04-0.36)**  **0.01** |
| FHA | **0.41**  **(0.22-0.57)**  **<0.01** | **0.25**  **(0.04-0.43)**  **0.02** | **0.52**  **(0.36-0.66)**  **<0.01** |  | **0.45**  **(0.31-0.58)**  **<0.01** | **0.64**  **(0.39-0.80)**  **<0.01** | **0.27**  **(0.11-0.42)**  **<0.01** | **0.17**  **(0.00-0.32)**  **0.05** | **0.26**  **(0.10-0.41)**  **<0.01** | **0.21**  **(0.04-0.36)**  **0.01** | **0.24**  **(0.08-0.39)**  **<0.01** | **0.28**  **(0.12-0.42)**  **<0.01** | **0.24**  **(0.07-0.39)**  **<0.01** | **0.24**  **(0.08-0.39)**  **<0.01** | **0.26**  **(0.10-0.41)**  **<0.01** | **0.23**  **(0.07-0.38)**  **<0.01** | 0.16  (-0.01-0.32)  0.06 | 0.12  (-0.04-0.28)  0.14 | **0.19**  **(-0.03-0.35)**  **0.02** | 0.00  (-16.5-16.6)  0.99 | 0.09  (-0.08-0.25)  0.29 | **0.17**  **(0.00-0.33)**  **0.05** |
| PRN | **0.45**  **(0.27-0.60)**  **<0.01** | **0.28**  **(0.07-0.46)**  **<0.01** | **0.25**  **(0.05-0.44)**  **0.02** | **0.51**  **(0.34-0.65)**  **<0.01** |  | **0.46**  **(0.15-0.69)**  **<0.01** | **0.25**  **(0.09-0.40)**  **<0.01** | **0.31**  **(0.15-0.45)**  **<0.01** | **0.31**  **(0.15-0.45)**  **<0.01** | **0.31**  **(0.15-0.45)**  **<0.01** | **0.32**  **(0.17-0.47)**  **<0.01** | **0.41**  **(0.26-0.53)**  **<0.01** | **0.34**  **(0.19-0.48)**  **<0.01** | **0.35**  **(0.20-0.49)**  **<0.01** | **0.44**  **(0.30-0.57)**  **<0.01** | **0.31**  **(0.16-0.46)**  **<0.01** | **0.26**  **(0.10-0.41)**  **<0.01** | **0.17**  **(0.01-0.33)**  **0.04** | **0.26**  **(0.10-0.41)**  **<0.01** | **0.11**  **(-0.05-0.27)**  **0.18** | **0.22**  **(0.06-0.38)**  **<0.01** | **0.29**  **(0.13-0.43)**  **<0.01** |
| Hib* | **0.32**  **(0.13-0.50)**  **<0.01** | **0.24**  **(0.04-0.43)**  **<0.01** | 0.04  (-0.17-0.25)  0.70 | **0.28**  **(0.07-0.46)**  **<0.01** | 0.14  (-0.07-0.34)  0.18 |  | 0.02  (-0.32-0.36)  0.91 | -0.07  (-0.40-0.28)  0.71 | -0.05  (-0.38-0.29)  0.77 | **0.43**  **(0.10-0.67)**  **0.01** | **0.60**  **(0.33-0.78)**  **<0.01** | **0.54**  **(0.25-0.74)**  **<0.01** | **0.38**  **(0.04-0.63)**  **0.02** | **0.45**  **(0.13-0.68)**  **<0.01** | **0.48**  **(0.17-0.70)**  **<0.01** | **0.42**  **(0.09-0.66)**  **0.01** | **0.61**  **(0.34-0.79)**  **<0.01** | 0.26  (-0.09-0.55)  0.14 | **0.46**  **(0.14-0.69)**  **<0.01** | 0.16  (-0.19-0.47)  0.37 | **0.35**  **(0.02-0.62)**  **0.04** | 0.20  (-0.14-0.51)  0.25 |
| IPV type 1 | **0.30**  **(0.10-0.47)**  **<0.01** | **0.28**  **(0.08-0.46)**  **<0.01** | 0.18  (-0.02-0.37)  0.08 | **0.24**  **(0.04-0.43)**  **0.02** | **0.44**  **(0.26-0.60)**  **<0.01** | 0.14  (-0.06-0.34)  0.18 |  | **0.57**  **(0.44-0.67)**  **<0.01** | **0.58**  **(0.46-0.68)**  **<0.01** | **0.17**  **(0.00-0.32)**  **0.05** | 0.12  (-0.04-0.28)  0.15 | **0.29**  **(0.13-0.43)**  **<0.01** | **0.17**  **(0.00-0.32)**  **0.05** | **0.20**  **(0.04-0.35)**  **0.02** | **0.25**  **(0.09-0.40)**  **<0.01** | **0.21**  **(0.04-0.36)**  **0.01** | 0.19  (0.03-0.35)  0.02 | **0.22**  **(0.06-0.37)**  **<0.01** | **0.22**  **(0.05-0.37)**  **<0.01** | 0.03  (-0.13-0.20)  0.68 | 0.09  (-0.08-0.25)  0.30 | 0.16  (-0.01-0.31)  0.06 |
| IPV type 2 | **0.25**  **(0.04-0.43)**  **0.02** | **0.21**  **(0.00-0.40)**  **0.05** | **0.24**  **(0.04-0.43)**  **0.02** | 0.18  (-0.02-0.37)  0.08 | **0.24**  **(0.03-0.43)**  **0.02** | 0.13  (-0.07-0.33)  0.21 | **0.71**  **(0.56-0.80)**  **<0.01** |  | **0.63**  **(0.51-0.72)**  **<0.01** | 0.11  (-0.05-0.28)  0.17 | **0.16**  **(0.00-0.32)**  **0.05** | **0.28**  **(0.12-0.42)**  **<0.01** | **0.19**  **(0.02-0.34)**  **0.03** | **0.20**  **(0.04-0.36)**  **0.02** | **0.19**  **(0.02-0.34)**  **0.03** | **0.19**  **(0.03-0.35)**  **0.02** | 0.15  (-0.02-0.30)  0.08 | 0.12  (-0.05-0.28)  0.16 | **0.20**  **(0.03-0.35)**  **0.02** | 0.02  (-0.14-0.19)  0.78 | 0.08  (-0.09-0.24)  0.34 | 0.10  (-0.07-0.26)  0.25 |
| IPV type 3 | **0.25**  **(0.10-0.47)**  **<0.01** | 0.14  (-0.06-0.34)  0.18 | 0.07  (-0.13-0.28)  0.49 | 0.08  (-0.13-0.28)  0.46 | **0.24**  **(0.03-0.42)**  **0.02** | 0.20  (0.00-0.39)  0.06 | **0.71**  **(0.59-0.80)**  **<0.01** | **0.74**  **(0.64-0.82)**  **<0.01** |  | 0.14  (-0.02-0.30)  0.09 | **0.20**  **(0.03-0.35)**  **0.02** | **0.31**  **(0.16-0.45)**  **<0.01** | **0.19**  **(0.02-0.34)**  **0.03** | **0.20**  **(0.04-0.36)**  **0.02** | 0.11  (-0.06-0.27)  0.20 | **0.17**  **(0.00-0.33)**  **0.04** | 0.15  (-0.01-0.31)  0.07 | 0.10  (-0.07-0.26)  0.26 | 0.13  (-0.03-0.29)  0.12 | 0.09  (-0.08-0.25)  0.30 | **0.08**  **(-0.09-0.24)**  **0.04** | 0.10  (-0.07-0.26)  0.24 |
| Pn 1 | **0.50**  **(0.33-0.64)**  **<0.01** | **0.31**  **(0.11-0.59)**  **<0.01** | 0.17  (-0.04-0.36)  0.11 | **0.22**  **(0.01-0.41)**  **0.04** | 0.20  (0.00-0.39)  0.06 | **0.40**  **(0.21-0.56)**  **<0.01** | 0.20  (0.00-0.39)  0.06 | **0.22**  **(0.01-0.41)**  **0.04** | 0.16  (-0.04-0.36)  0.12 |  | **0.54**  **(0.41-0.65)**  **<0.01** | **0.64**  **(0.53-0.73)**  **<0.01** | **0.74**  **(0.66-0.81)**  **<0.01** | **0.65**  **(0.55-0.74)**  **<0.01** | **0.56**  **(0.44-0.67)**  **<0.01** | **0.64**  **(0.53-0.73)**  **<0.01** | **0.64**  **(0.53-0.73)**  **<0.01** | **0.35**  **(0.20-0.49)**  **<0.01** | **0.63**  **(0.52-0.72)**  **<0.01** | **0.41**  **(0.26-0.54)**  **<0.01** | **0.39**  **(0.24-0.52)**  **<0.01** | **0.56**  **(0.44-0.67)**  **<0.01** |
| Pn 3 | **0.28**  **(0.07-0.46)**  **<0.01** | **0.25**  **(0.05-0.43)**  **0.02** | 0.16  (-0.04-0.35)  0.12 | 0.19  (-0.02-0.38)  0.07 | 0.11  (-0.10-0.31)  0.31 | **0.28**  **(0.08-0.46)**  **<0.01** | 0.20  (0.00-0.39)  0.06 | **0.23**  **(0.02-0.41)**  **0.03** | **0.23**  **(0.03-0.42)**  **0.03** | **0.61**  **(0.46-0.72)**  **<0.01** |  | **0.62**  **(0.50-0.71)**  **<0.01** | **0.48**  **(0.34-0.60)**  **<0.01** | **0.43**  **(0.29-0.56)**  **<0.01** | **0.39**  **(0.24-0.52)**  **<0.01** | **0.48**  **(0.34-0.60)**  **<0.01** | **0.55**  **(0.42-0.66)**  **<0.01** | **0.20**  **(0.37.0.35)**  **<0.01** | **0.59**  **(0.47-0.69)**  **<0.01** | **0.36**  **(0.21-0.49)**  **<0.01** | **0.34**  **(0.19-0.48)**  **<0.01** | **0.40**  **(0.25-0.53)**  **<0.01** |
| Pn 4 | **0.46**  **(0.28-0.61)**  **<0.01** | **0.24**  **(0.03-0.42)**  **0.02** | 0.08  (-0.12-0.28)  0.43 | 0.15  (-0.06-0.34)  0.17 | 0.17  (-0.04-0.36)  0.11 | **0.27**  **(0.07-0.45)**  **0.01** | **0.23**  **(0.02-0.41)**  **0.03** | **0.24**  **(0.04-0.42)**  **0.02** | **0.32**  **(0.12-0.49)**  **<0.01** | **0.78**  **(0.78-0.85)**  **<0.01** | **0.58**  **(0.43-0.70)**  **<0.01** |  | **0.69**  **(0.59-0.77)**  **<0.01** | **0.58**  **(0.46-0.68)**  **<0.01** | **0.52**  **(0.39-0.63)**  **<0.01** | **0.63**  **(0.52-0.72)**  **<0.01** | **0.62**  **(0.51-0.72)**  **<0.01** | **0.44**  **(0.30-0.57)**  **<0.01** | **0.62**  **(0.51-0.71)**  **<0.01** | **0.35**  **(0.20-0.49)**  **<0.01** | **0.43**  **(0.28-0.55)**  **<0.01** | **0.44**  **(0.30-0.57)**  **<0.01** |
| Pn 5 | **0.51**  **(0.33-0.64)**  **<0.01** | **0.34**  **(0.14-0.51)**  **<0.01** | **0.21**  **(0.00-0.40)**  **0.04** | **0.21**  **(0.01-0.40)**  **0.04** | **0.20**  **(0.00-0.39)**  **0.05** | **0.37**  **(0.18-0.54)**  **<0.01** | **0.25**  **(0.05-0.43)**  **0.02** | **0.27**  **(0.07-0.45)**  **<0.01** | **0.24**  **(0.04-0.43)**  **0.02** | **0.86**  **(0.79-0.91)**  **<0.01** | **0.55**  **(0.39-0.68)**  **<0.01** | **0.79**  **(0.69-0.85)**  **<0.01** |  | **0.69**  **(0.59-0.77)**  **<0.01** | **0.61**  **(0.49-0.70)**  **<0.01** | **0.75**  **(0.66-0.81)**  **<0.01** | **0.73**  **(0.64-0.80)**  **<0.01** | **0.50**  **(0.37-0.62)**  **<0.01** | **0.70**  **(0.60-0.77)**  **<0.01** | **0.44**  **(0.30-0.56)**  **<0.01** | **0.48**  **(0.34-0.60)**  **<0.01** | **0.54**  **(0.41-0.64)**  **<0.01** |
| Pn 6A | **0.51**  **(0.34-0.65)**  **<0.01** | 0.17  (-0.03-0.36)  0.11 | 0.13  (-0.08-0.33)  0.21 | 0.12  (-0.09-0.31)  0.27 | 0.10  (-0.10-0.30)  0.33 | **0.30**  **(0.10-0.48)**  **<0.01** | 0.08  (-0.12-0.28)  0.43 | 0.17  (-0.03-0.37)  0.10 | 0.08  (-0.12-0.28)  0.46 | **0.78**  **(0.69-0.85)**  **<0.01** | **0.49**  **(0.31-0.63)**  **<0.01** | **0.67**  **(0.54-0.77)**  **<0.01** | **0.73**  **(0.61-0.81)**  **<0.01** |  | **0.77**  **(0.69-0.83)**  **<0.01** | **0.66**  **(0.55-0.74)**  **<0.01** | **0.62**  **(0.51-0.72)**  **<0.01** | **0.40**  **(0.25-0.53)**  **<0.01** | **0.58**  **(0.46-0.68)**  **<0.01** | **0.28**  **(0.12-0.42)**  **<0.01** | **0.39**  **(0.24-0.52)**  **<0.01** | **0.59**  **(0.47-0.69)**  **<0.01** |
| Pn 6B | **0.62**  **(0.47-0.73)**  **<0.01** | 0.15  (-0.05-0.35)  0.14 | 0.20  (-0.01-0.39)  0.06 | 0.15  (-0.05-0.35)  0.15 | 0.19  (-0.02-0.38)  0.07 | **0.32**  **(0.12-0.49)**  **<0.01** | **0.25**  **(0.05-0.43)**  **0.02** | **0.31**  **(0.11-0.48)**  **<0.01** | **0.25**  **(0.04-0.43)**  **0.02** | **0.67**  **(0.54-0.77)**  **<0.01** | **0.36**  **(0.17-0.53)**  **<0.01** | **0.62**  **(0.47-0.73)**  **<0.01** | **0.61**  **(0.46-0.72)**  **<0.01** | **0.76**  **(0.66-0.84)**  **<0.01** |  | **0.58**  **(0.46-0.68)**  **<0.01** | **0.58**  **(0.46-0.68)**  **<0.01** | **0.39**  **(0.24-0.52)**  **<0.01** | **0.55**  **(0.42-0.65)**  **<0.01** | **0.32**  **(0.17-0.47)**  **<0.01** | **0.43**  **(0.29-0.56)**  **<0.01** | **0.68**  **(0.58-0.76)**  **<0.01** |
| Pn 7F | **0.52**  **(0.36-0.66)**  **<0.01** | **0.29**  **(0.09-0.47)**  **<0.01** | 0.12  (-0.09-0.32)  0.27 | 0.20  (-0.01-0.29)  0.06 | 0.17  (-0.03-0.37)  0.10 | **0.29**  **(0.09-0.47)**  **<0.01** | 0.14  (-0.07-0.33)  0.20 | 0.18  (-0.03-0.37)  0.09 | 0.14  (-0.07-0.33)  0.20 | **0.78**  **(0.69-0.85)**  **<0.01** | **0.55**  **(0.38-0.68)**  **<0.01** | **0.70**  **(0.57-0.79)**  **<0.01** | **0.73**  **(0.62-0.81)**  **<0.01** | **0.75**  **(0.65-0.83)**  **<0.01** | **0.62**  **(0.48-0.73)**  **<0.01** |  | **0.71**  **(0.61-0.78)**  **<0.01** | **0.41**  **(0.26-0.54)**  **<0.01** | **0.67**  **(0.57-0.75)**  **<0.01** | **0.33**  **(0.17-0.47)**  **<0.01** | **0.38**  **(0.23-0.51)**  **<0.01** | **0.53**  **(0.40-0.64)**  **<0.01** |
| Pn 9V | **0.52**  **(0.36-0.66)**  **<0.01** | **0.28**  **(0.07-0.46)**  **<0.01** | **0.26**  **(0.06-0.44)**  **0.01** | **0.25**  **(0.05-0.44)**  **0.01** | **0.26**  **(0.05-0.44)**  **0.01** | 0.20  (-0.01-0.39)  0.06 | 0.16  (-0.05-0.35)  0.14 | 0.17  (-0.03-0.37)  0.10 | 0.16  (-0.05-0.35)  0.13 | **0.85**  **(0.78-0.90)**  **<0.01** | **0.55**  **(0.38-0.68)**  **<0.01** | **0.78**  **(0.69-0.85)**  **<0.01** | **0.78**  **(0.68-0.85)**  **<0.01** | **0.69**  **(0.57-0.79)**  **<0.01** | **0.58**  **(0.43-0.70)**  **<0.01** | **0.75**  **(0.64-0.83)**  **<0.01** |  | **0.36**  **(0.21-0.50)**  **<0.01** | **0.69**  **(0.59-0.77)**  **<0.01** | **0.40**  **(0.24-0.53)**  **<0.01** | **0.38**  **(0.23-0.51)**  **<0.01** | **0.51**  **(0.37-0.62)**  **<0.01** |
| Pn 14 | **0.31**  **(0.11-0.49)**  **<0.01** | 0.05  (-0.16-0.25)  0.66 | 0.13  (-0.08-0.33)  0.21 | 0.03  (-0.18-0.23)  0.78 | 0.12  (-0.08-0.32)  0.24 | 0.06  (-0.14-0.26)  0.57 | 0.13  (-0.08-0.33)  0.22 | 0.11  (-0.10-0.31)  0.31 | **0.23**  **(0.03-0.42)**  **0.03** | **0.52**  **(0.36-0.66)**  **<0.01** | **0.39**  **(0.20-0.55)**  **<0.01** | **0.50**  **(0.33-0.64) <0.01** | **0.44**  **(0.25-0.59)**  **<0.01** | **0.54**  **(0.38-0.67)**  **<0.01** | **0.46**  **(0.28-0.61)**  **<0.01** | **0.52**  **(0.36-0.66)**  **<0.01** | **0.52**  **(0.35-0.65)**  **<0.01** |  | **0.33**  **(0.18-0.47)**  **<0.01** | **0.27**  **(0.11-0.42)**  **<0.01** | **0.30**  **(0.14-0.44)**  **<0.01** | **0.33**  **(0.18-0.47)**  **<0.01** |
| Pn 18C | **0.49**  **(0.32-0.63)**  **<0.01** | 0.18  (-0.03-0.37)  0.09 | 0.09  (-0.12-0.29)  0.41 | 0.15  (-0.06-0.34)  0.16 | 0.16  (-0.05-0.36)  0.13 | **0.41**  **(0.22-0.57)**  **<0.01** | **0.22**  **(0.01-0.41)**  **0.04** | **0.22**  **(0.01-0.41)**  **0.04** | **0.28**  **(0.08-0.46)**  **<0.01** | **0.80**  **(0.71-0.86)**  **<0.01** | **0.62**  **(0.48.0.74)**  **<0.01** | **0.78**  **(0.69-0.85)**  **<0.01** | **0.78**  **(0.69-0.85)**  **<0.01** | **0.74**  **(0.63.0.82)**  **<0.01** | **0.67**  **(0.54-0.77)**  **<0.01** | **0.76**  **(0.65-0.83)**  **<0.01** | **0.70**  **(0.58-0.80)**  **<0.01** | **0.62**  **(0.47-0.73)**  **<0.01** |  | **0.43**  **(0.28-0.56)**  **<0.01** | **0.48**  **(0.34-0.60)**  **<0.01** | **0.53**  **(0.40-0.64)**  **<0.01** |
| Pn 19A | **0.53**  **(0.37-0.67)**  **<0.01** | 0.18  (-0.02-0.37)  0.08 | 0.12  (-0.08-0.32)  0.24 | 0.14  (-0.07-0.34)  0.18 | **0.23**  **(0.03-0.42)**  **0.03** | **0.25**  **(0.05-0.44)**  **0.01** | **0.33**  **(0.13-0.50)**  **<0.01** | **0.29**  **(0.09-0.47)**  **<0.01** | **0.39**  **(0.20-0.55)**  **<0.01** | **0.66**  **(0.52-0.76)**  **<0.01** | **0.49**  **(0.32-0.64)**  **<0.01** | **0.67**  **(0.54-0.77)**  **<0.01** | **0.64**  **(0.49-0.74)**  **<0.01** | **0.66**  **(0.52-0.76)**  **<0.01** | **0.63**  **(0.48-0.74)**  **<0.01** | **0.59**  **(0.44-0.71)**  **<0.01** | **0.64**  **(0.50-0.75)**  **<0.01** | **0.55**  **(0.38-0.68)**  **<0.01** | **0.74**  **(0.63-0.82)**  **<0.01** |  | **0.75**  **(0.71-0.82)**  **<0.01** | **0.39**  **(0.24-0.52)**  **<0.01** |
| Pn 19F | **0.57**  **(0.41-0.69)**  **<0.01** | **0.26**  **(0.05-0.44)**  **0.01** | **0.25**  **(0.05-0.43)**  **0.02** | **0.26**  **(0.05-0.44)**  **0.01** | **0.34**  **(0.14-0.51)**  **<0.01** | **0.33**  **(0.13-0.50)**  **<0.01** | **0.23**  **(0.03-0.42)**  **0.03** | 0.19  (-0.01-0.38)  0.07 | **0.21**  **(0.00-0.40)**  **0.04** | **0.75**  **(0.64-0.83)**  **<0.01** | **0.51**  **(0.34-0.65)**  **<0.01** | **0.66**  **(0.53-0.77)**  **<0.01** | **0.71**  **(0.59-0.80)**  **<0.01** | **0.69**  **(0.56-0.78)**  **<0.01** | **0.66**  **(0.52-0.76)**  **<0.01** | **0.72**  **(0.61-0.81)**  **<0.01** | **0.76**  **(0.66-0.84)**  **<0.01** | **0.58**  **(0.43-0.70)**  **<0.01** | **0.75**  **(0.65-0.83)**  **<0.01** | **0.73**  **(0.61-0.81)**  **<0.01** |  | **0.30**  **(0.14-0.45)**  **<0.01** |
| Pn 23F | **0.56**  **(0.40-0.68)**  **<0.01** | **0.30**  **(0.10-0.48)**  **<0.01** | 0.17  (-0.04-0.36)  0.11 | **0.24**  **(0.04-0.43)**  **0.02** | **0.24**  **(0.04-0.43)**  **0.02** | **0.33**  **(0.14-0.50)**  **<0.01** | **0.21**  **(0.00-0.40)**  **0.05** | **0.27**  **(0.07-0.45)**  **<0.01** | **0.25**  **(0.04-0.43)**  **0.02** | **0.79**  **(0.70-0.85)**  **<0.01** | **0.59**  **(0.44-0.71)**  **<0.01** | **0.75**  **(0.64-0.83)**  **<0.01** | **0.78**  **(0.68-0.85)**  **<0.01** | **0.78**  **(0.69-0.85)**  **<0.01** | **0.70**  **(0.57-0.79)**  **<0.01** | **0.75**  **(0.65-0.83)**  **<0.01** | **0.75**  **(0.64-0.83)**  **<0.01** | **0.49**  **(0.31-0.63)**  **<0.01** | **0.77**  **(0.67-0.84)**  **<0.01** | **0.75**  **(0.64-0.83)**  **<0.01** | **0.75**  **(0.64-0.83)**  **<0.01** |  |

*seven months after vaccination only participants who had not had MMR/Hib-MenC

**Supplementary Table 2** Correlation (expressed as Spearman correlation coefficients with 95% confidence interval and p-values) for pairwise comparisons of antibody responses to the12-month vaccinations measured one month after vaccination

|  | Measles | Mumps | Rubella | MenC | Tetanus | Hib |
| --- | --- | --- | --- | --- | --- | --- |
|  |  |  |  |  |  |  |
| Measles |  | **0.20**  **(0.04-0.35)**  **0.01** | **0.17**  **(0.01-0.33)**  **0.04** | 0.07  (-0.09-0.23)  0.39 | -0.09  (-0.24-0.08)  0.30 | -0.13  (-0.28-0.04)  0.13 |
| Mumps |  |  | **0.61**  **(0.50-0.70)**  **<0.01** | -0.06  (-0.22-0.10)  0.45 | 0.05  (-0.11-0.21)  0.56 | -0.12  (-0.28-0.04)  0.14 |
| Rubella |  |  |  | -0.09  (-0.25-0.07)  0.25 | -0.07  (-0.23-0.09)  0.38 | -0.13  (-0.28-0.03)  0.12 |
| MenC |  |  |  |  | **0.52**  **(0.39-0.63)**  **<0.01** | **0.48**  **(0.35-0.60)**  **<0.01** |
| Tetanus |  |  |  |  |  | **0.50**  **(0.36-0.61)**  **<0.01** |
